# Modulation of Cell Metabolic Pathways and Oxidative Stress Signaling Contribute to Acquired Melphalan Resistance in Multiple Myeloma Cells

**DOI:** 10.1371/journal.pone.0119857

**Published:** 2015-03-13

**Authors:** Kamila Anna Zub, Mirta Mittelstedt Leal de Sousa, Antonio Sarno, Animesh Sharma, Aida Demirovic, Shalini Rao, Clifford Young, Per Arne Aas, Ida Ericsson, Anders Sundan, Ole Nørregaard Jensen, Geir Slupphaug

**Affiliations:** 1 Department of Cancer Research and Molecular Medicine, Norwegian University of Science and Technology NTNU, Trondheim, Norway; 2 PROMEC Core Facility for Proteomics and Metabolomics, Norwegian University of Science and Technology NTNU, Trondheim, Norway; 3 Department of Biochemistry and Molecular Biology, University of Southern Denmark, Odense, Denmark; University of South Alabama, UNITED STATES

## Abstract

Alkylating agents are widely used chemotherapeutics in the treatment of many cancers, including leukemia, lymphoma, multiple myeloma, sarcoma, lung, breast and ovarian cancer. Melphalan is the most commonly used chemotherapeutic agent against multiple myeloma. However, despite a 70–80% initial response rate, virtually all patients eventually relapse due to the emergence of drug-resistant tumour cells. By using global proteomic and transcriptomic profiling on melphalan sensitive and resistant RPMI8226 cell lines followed by functional assays, we discovered changes in cellular processes and pathways not previously associated with melphalan resistance in multiple myeloma cells, including a metabolic switch conforming to the Warburg effect (aerobic glycolysis), and an elevated oxidative stress response mediated by VEGF/IL8-signaling. In addition, up-regulated aldo-keto reductase levels of the AKR1C family involved in prostaglandin synthesis contribute to the resistant phenotype. Finally, selected metabolic and oxidative stress response enzymes were targeted by inhibitors, several of which displayed a selective cytotoxicity against the melphalan-resistant cells and should be further explored to elucidate their potential to overcome melphalan resistance.

## Introduction

Multiple myeloma (MM) is an incurable bone marrow disease and the second most common hematological cancer. The median age of onset is 65 years and progression often leads to severe complications including immunodeficiency, osteolytic bone disease and renal failure [1]. Although current therapies may improve the patient’s survival, disease progression and acquired drug resistance remain unsolved issues. Since the 1960s, the alkylating drug melphalan (L-phenylalanine mustard) has been employed in combination with corticosteroids as first-line therapy for MM [2]. Novel agents such as bortezomib and lenalidomide have recently been introduced, but melphalan remains the standard therapy for transplant-ineligible patients and is the basis for high-dose therapy associated with autologous stem cell transplant [3]. Melphalan’s efficacy has been attributed to its ability to induce cytotoxic interstrand cross-links (ICLs) in DNA [4], but it may also induce other lesions in DNA [5], RNA, proteins and lipids [6]. The mechanisms by which melphalan kills tumor cells thus remain elusive and identifying factors that attenuate melphalan sensitivity is crucial to improving therapeutic outcomes.

Acquired melphalan resistance in MM has been associated with reduced drug uptake [7], increased drug detoxification [8,9], reduced ICL formation and enhanced DNA repair of ICL lesions [10–12], modulation of DNA base excision and strand break repair [13,14], adaptation to reactive oxygen species (ROS) [15] and decreased apoptosis [16]; however, there are no robust biomarkers that predict melphalan resistance.

Here we have used transcriptomics and proteomics to investigate cellular changes associated with acquired melphalan resistance in the RPMI8226 multiple myeloma cell line. We observed a metabolic switch conforming to the Warburg effect in the melphalan-resistant cell line accompanied by an increased oxidative stress response and enhanced survival and proliferation signaling. The increased survival was partially mediated through VEGF- and IL8-induced PI3K/p38 signaling and upregulated expression of the AKR1C family of aldo-keto reductases. We demonstrate that targeting enzymes within the affected pathways by specific inhibitors can overcome acquired melphalan resistance.

## Materials and Methods

### Reagents and antibodies

For Western analysis antibodies to AKR1C2 (H00001646-D01, Abnova), AKR1C3 (H00008644-B01, Abnova), AKR1C4 (H00001109-M01, Novus), AKT1 (#2967, Cell Signaling), Caspase3 (sc-7148, Santa Cruz), SLC16A3 (OAAB08662, Aviva Systems Biology) PARP-1 (sc-74470, Santa Cruz), STAT3 (sc-81385, Santa Cruz), pSTAT3 (S2690, Sigma) and β-actin (ab8226, Abcam) primary antibodies and HRP-conjugated secondary antibodies (Dako) were used. Melphalan, ursodeoxyholate, indomethacin, flufenamic acid, dichloroacetic acid, 2-deoxy-D-glucose, sodium oxamate, metformin, oligomycin, antimycinA, FLLL31, wortmannin, rapamycin, methyl glyoxal, acetylsalicylic acid, ibuprofen, (Sigma Aldrich), tert-butyl peroxide (Fluka), LY294002, SB203580 and BIRB0796 (Cell Signaling) were used in viability assays.

### Cell lines and preparation of cell extracts

MM cell lines RPMI8226 and RPMI8226-LR5 were kindly donated by Prof. William S. Dalton at the H. Lee Moffitt Cancer Center & Research Institute, Tampa, USA. Cells were maintained, treated with melphalan and cell extracts prepared as previously described [13].

### mRNA isolation and analysis

mRNA was isolated from six batches each of control and melphalan-treated RPMI8226 and RPMI8226-LR5 cells using the mirVana mRNA isolation kit (Ambion). RNA concentration and quality were determined using NanoDrop ND-8000 (NanoDrop Technologies) and Agilent 2100 Bioanalyzer (Agilent). RIN values of RNA samples used for cRNA amplification ranged from 8.5 to 10 (mean = 9.49). The Illumina TotalPrep RNA amplification Kit (Ambion) was used to amplify mRNA for hybridization on Illumina BeadChips. First strand cDNA was synthesized from each mRNA sample. Following second strand synthesis and purification, cRNA was synthesized for 12 hours. Gene expression profiles were measured using Illumina HumanHT-12_V3 Expression BeadChip. Raw data were exported from Illumina GenomeStudio to R using the Bioconductor package `lumi`version 2.1.3. [17]. Data were quantile normalized and log_2_ transformed. The groups were compared using a t-test with empirical Bayes correction from the Bioconductor package `Limma`[18]. The fold change was used to demonstrate rate of changes in average gene expressions between studied groups. Statistical analyses were performed using the false discovery rate (FDR) with a significance threshold of 0.01. The transcriptomic data have been deposited to the GEO repository with the identifier GSE60970 [19].

### Western analysis, viability assay and mROS analysis

Western analysis and MTT assays were performed as described [13]. mROS was analyzed using MitoSOX Red (Molecular Probes) according to the manufacturer’s protocol. Briefly, cells (0.5 × 10^6^ cells/ml) were pretreated with inhibitors for 6 h, washed with HBBS and incubated with MitoSOX for 10 min at 37°C. Flow cytometry was performed at 510 nm excitation and 580 nm emissions.

### Cytokine and lactate analysis

Briefly cells (0.5 × 10^6^ cells/ml) were incubated in RPMI1640 medium until they reached approximately 1 × 10^6^ cells/ml and 80% viability. Medium (50 μl) was used for cytokine measurements in duplicates using the Bioplex 27-plex human cytokine kit from BioRad as per manufacturer's instructions. Bioplex manager software was used for calculation of cytokine concentrations. Standard curves were optimized by the software and verified manually. Lactate levels were measured using the L-Lactate Assay Kit according to the manufacturer’s protocol (Cayman). Extracellular lactate level was measured in medium, intracellular lactate in 15 × 10^6^ cells. Results were normalized and are shown as concentration of lactate/1 × 10^6^ cells.

### Stable isotope labeling with amino acids in cell culture (SILAC) and LC-MS/MS analysis

RPMI8226 and RPMI8226-LR5 cells were grown in light (^12^C6-lysine) and heavy (^13^C6-lysine) medium, respectively (Pierce SILAC protein Quantitation kit-RPMI1640). Sensitive and resistant cells were mixed 1:1 and cell extracts prepared as above. Proteins were separated on 4–12% denaturing NuPAGE (Invitrogen) and stained with Simply Blue^TM^ (Invitrogen). Thirteen bands were excised, reduced and alkylated prior to trypsin in-gel digestion as described [20]. Peptides were eluted with 50% acetonitrile in 5% formic acid and lyophilized before MS analysis. LC-MS/MS was conducted on an EASY-nLC (Proxeon) coupled to an LTQ-Orbitrap XL mass spectrometer (Thermo). Reconstituted samples were separated on an in-house 15 cm fused silica column (100 μm i.d., 375 μm o.d.) using a 50 min gradient up to 32% ACN in 0.1% formic acid and 250 nl/min flow rate. MS scans (300–1800 m/z) were acquired at a resolution of 60,000 (m/z 400) in the Orbitrap. During each duty cycle, up to five of the most intense peptides were selected for CID fragmentation in the ion trap. Raw files were processed in MaxQuant v.1.0.13.8, with Mascot searches utilizing human IPI database (version 3.52) containing extra entries for reverse protein sequences. Search criteria included carbamidomethylation as fixed modification, deamidation (NQ) and oxidation (M) as variable modifications, maximum of two missed cleavage sites and MS/MS tolerance of 0.6 Da. FDR thresholds for peptide and protein identifications were set to 1%. A minimum of two unique peptides was specified for protein identifications, with protein quantification requiring at least two ratio measurements. The proteomics data have been deposited to the ProteomeXchange Consortium [21] via the PRIDE partner repository with the identifier PXD001276 [22].

### Statistical analysis

Significance and p-value in viability assays were obtained from the GraphPad Prism software (GraphPad Software). Each experiment was conducted in triplicate, data were analyzed using one-way ANOVA and student—Newman-Keuls multiple test, where: ns—p>0.05, *- 0.05≥p>0.01, **- 0.01≥p>0.001, *** - p≤ 0.001.

## Results

### Illumina and SILAC gene expression profiling

**Fig 1:**
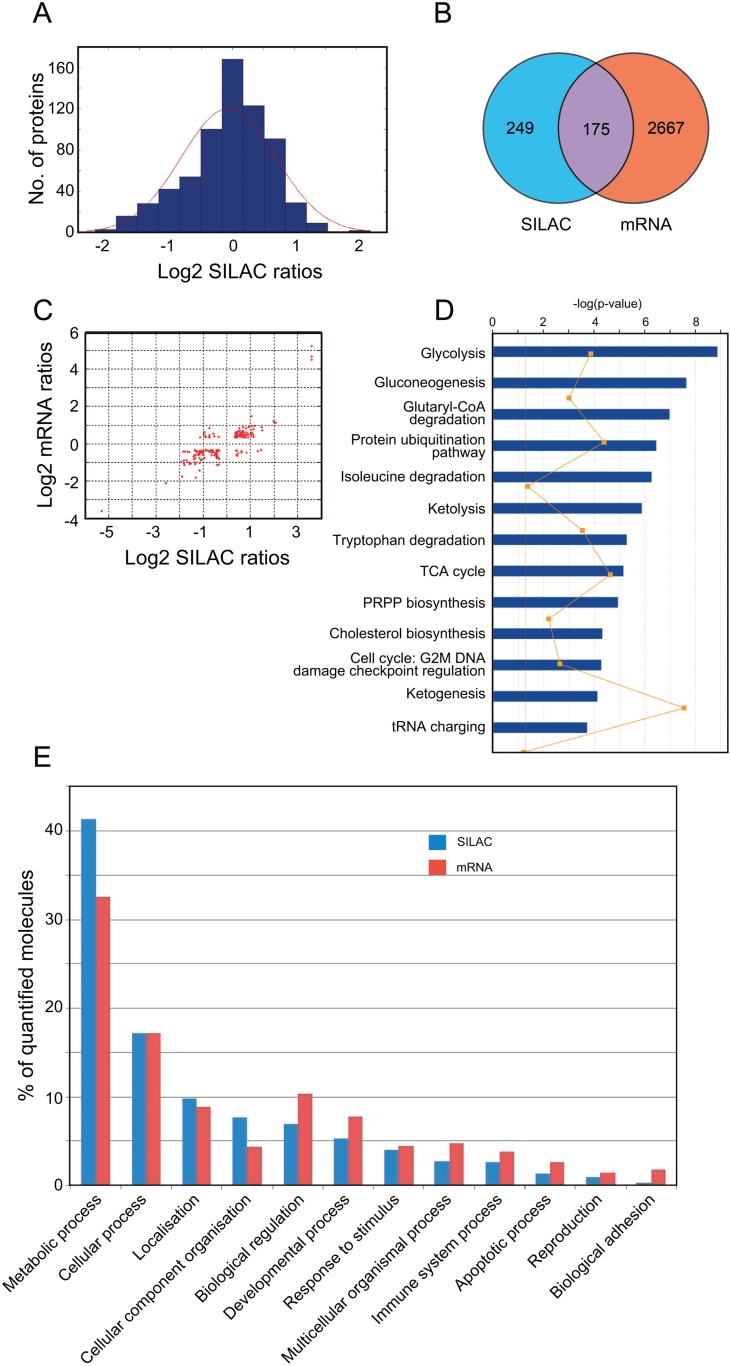
SILAC and mRNA quantitative profiling. (A), histogram of log_2_ SILAC ratios. (B), Venn diagram of DEGs identified in SILAC and mRNA analyses (threshold 1.25 fold change). (C), Scatter plot illustrating fold change relationships between DEGs identified in SILAC and mRNA analyses. (D), Most affected canonical pathways as identified by IPA analysis of SILAC data (threshold 1.25x change). The orange line represents the ratio of the number of genes represented within each pathway to the total number of genes in the pathway. (E), Distribution of DEGs in GO: biological process categories.

To investigate large scale alterations in gene expression accompanying melphalan resistance, we used the multiple myeloma cell line RPMI8226 and its melphalan-resistant derivative LR5. The stable isotope labeling by amino acids in cell culture (SILAC) approach resulted in the identification of 1042 proteins, of which 668 were quantified using MaxQuant (PEP <0.01) (S1 Table). The histogram of the log_2_ SILAC ratios showed a normal distribution centered at zero, supporting similar overall protein loads of the H/L samples (Fig. 1A). Illumina HumanHT-12_V3 annotation data were used for mapping mRNA expression and led to 4580 identifications with p-values < 0.05 (S2 Table). We considered differentially expressed genes (DEGs) exhibiting an absolute fold change above 1.25 because the cell lines were isogenic and proliferated similarly. This resulted in 424 and 2842 unique proteins and mRNAs, respectively (S1and S2 Tables), of which 175 proteins and mRNAs overlapped (Fig. 1B). We observed a high correlation of expression data from the protein and mRNA analyses (up-regulated R^2^ = 0.91, down-regulated R^2^ = 0.72, Fig. 1C). The most differentially regulated proteins in the SILAC analyses are listed in Table 1 together with their corresponding fold changes at the mRNA level.

**Table 1:** Most up- and downregulated genes in melphalan-resistant cells as determined from SILAC and Illumina analysis.

Gene symbol	Protein name	REFSEQ	Fold change SILAC^a^	Fold change Illumina^a^
AKR1C3	aldo-keto reductase family 1, member C3	NP_003730	**12,053**	**25,507**
VIM	vimentin	NP_003371	**4,212**	**2,208**
MACF1	microtubule-actin crosslinking factor 1	NP_149033	**3,968**	**2,263**
POLR1A	polymerase (RNA) I polypeptide A, 194kDa	NP_056240	**3,345**	
CBR1	carbonyl reductase 1	NP_001748	**2,839**	1,159
UAP1	UDP-N-acteylglucosamine pyrophosphorylase 1	NP_003106	**2,836**	**1,654**
KHDRBS1	KH domain containing, RNA bindingSAM68	NP_006550	**2,796**	1,193
ANXA5	annexin A5	NP_001145	**2,789**	**1,859**
G3BP1	GTPase activating protein (SH3 domain) binding protein 1	NP_005745	**2,759**	-1,192
KPNA2	karyopherin alpha 2 (RAG cohort 1, importin alpha 1)	NP_002257	**2,549**	1,093
PRPF19	PRP19/PSO4 pre-mRNA proc.factor 19	NP_055317	**2,483**	-1,311
PSME3	proteasome (prosome, macropain) activator subunit 3	NP_789839	**2,441**	1,169
ARD1A	ARD1 homolog A, N-acetyltransferase (S. cerevisiae)	NP_003482	**2,307**	1,212
HNRNPA3	heterogeneous nuclear ribonucleoprotein A3	NP_919223	**2,272**	**1,945**
HSPA8	heat shock 70kDa protein 8	NP_006588	**2,237**	1,158
XPO5	exportin 5	NP_065801	**2,199**	**1,287**
ABCE1	ATP-binding cassette, sub-family E (OABP), member 1	NP_001035809	**2,177**	**1,721**
PPID	peptidylprolyl isomerase D	NP_005029	**2,162**	-1,015
ACTN4	actinin, alpha 4	NP_004915	**2,156**	**1,396**
CKAP5	cytoskeleton associated protein 5	NP_001008938	**2,136**	1,237
G6PD	glucose-6-phosphate dehydrogenase	NP_000393	**2,101**	**1,935**
DDX19A	DEAD (Asp-Glu-Ala-As) box polypeptide 19A	NP_060802	**2,088**	**1,467**
NAPG	splicing factor proline/glutamine-rich	NP_003817	**2,054**	**2,813**
MYO1C	N-ethylmaleimide-sensitive factor attachment protein γ	NP_001074248	**2,025**	
PTBP1	polypyrimidine tract binding protein 1	NP_002810	**2,022**	1,328
CBR3	carbonyl reductase 3	NP_001227	**2,019**	**1,430**
LRBA	LPS-responsive vesicle trafficking, beach and anchor c.	NP_006717	**2,007**	**1,670**
RRBP1	ribosome binding protein 1 homolog 180kDa (dog)	NP_001036041	**-2,618**	**-1,773**
SERPINH1	serpin peptidase inhibitor, clade H member 1	NP_001226	**-2,639**	-1,609
MESDC2	mesoderm development candidate 2	NP_055969	**-2,733**	
FN1	fibronectin 1	NP_997647	**-2,849**	
SFN	stratifin	NP_006133	**-2,859**	**-2,069**
PCK2	phosphoenolpyruvate carboxykinase 2 (mitochondrial)	NP_004554	**-2,894**	-1,22
NSDHL	NAD(P) dependent steroid dehydrogenase-like	NP_001123237	**-2,904**	-1,155
DCI	dodecenoyl-Coenzyme A delta isomerase	NP_001910	**-2,956**	**-2,122**
MAN2A1	mannosidase, alpha, class 2A, member 1	NP_002363	**-3,007**	**-1,378**
STT3B	STT3, subunit of oligosaccharyltransferase compl. hom. B	NP_849193	**-3,016**	**-1,356**
PDIA5	protein disulfide isomerase family A, member 5	NP_006801	**-3,019**	**-1,946**
DNAJB11	DnaJ (Hsp40) homolog, subfamily B, member 11	NP_057390	**-3,045**	-1,130
ERP44	endoplasmic reticulum protein 44	NP_055866	**-3,070**	
DDOST	dolichyl-diphosphooligosaccharide-protein glycosyltransf.	NP_005207	**-3,163**	**-1,347**
PDIA4	protein disulfide isomerase family A, member 4	NP_004902	**-3,271**	**-1,835**
PDIA6	protein disulfide isomerase family A, member 6	NP_005733	**-3,419**	**-2,008**
ERP29	endoplasmic reticulum protein 29	NP_006808	**-3,487**	**-2,231**
IGF2R	insulin-like growth factor 2 receptor	NP_000867	**-3,530**	1,248
STAT1	signal transducer and activator of transcription 1, 91kDa	NP_009330	**-3,536**	-1,883
ASS1	argininosuccinate synthetase 1	NP_000041	**-3,543**	-2,069
STOML2	stomatin (EPB72)-like 2	NP_038470	**-3,594**	**-1,335**
CKB	creatine kinase, brain	NP_001814	**-3,615**	-1,126
CANX	calnexin	NP_001019820	**-3,675**	**-3,408**
IDH2	isocitrate dehydrogenase 2 (NADP+), mitochondrial	NP_002159	**-3,819**	**-1,977**
EPHX1	epoxide hydrolase 1, microsomal (xenobiotic)	NP_000111	**-3,983**	-1,13
PHGDH	phosphoglycerate dehydrogenase	NP_006614	**-6,014**	**-4,343**
C2orf30	endoplasmic reticulum lectin 1	NP_056516	**-8,235**	-1,340
PDIA2	protein disulfide isomerase family A, member 2	NP_006840	**-14,947**	
MYO6	myosin VI	NP_004990	**-20,807**	
MX1	myxovirus resistance 1, interferon-inducible protein p78	NP_002453	**-39,202**	**-12,073**

^a^Statistically significant values are highlighted in bold.

### Melphalan-resistant RPMI8226 cells display altered expression of metabolic enzymes conforming to the Warburg effect

**Fig 2:**
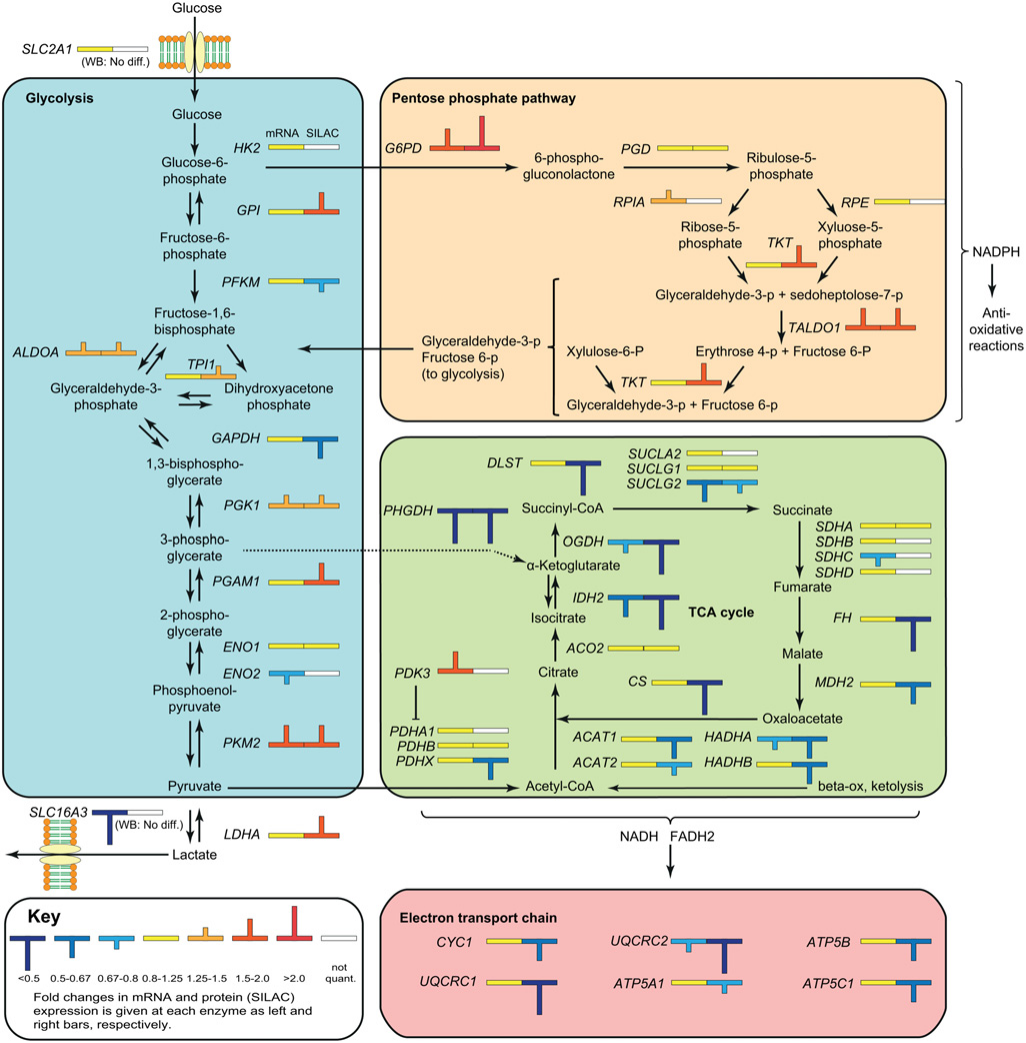
Overview of DEGs in the glycolytic and oxidative metabolic pathways. The observed up-regulation of factors in the glycolytic- and pentose phosphate pathways and down-regulation of factors in the TCA cycle and the mitochondrial electron transport chain conform to a Warburg type metabolic switch.

To search for modified biological pathways in the resistant cells, gene identifiers in the SILAC dataset were mapped in the Ingenuity Knowledge Base, entered into Ingenuity Pathway Analysis (IPA) and plotted onto canonical pathways. Glycolysis was identified as the most significantly altered pathway (p = 1.31 × 10^-9^), and several other respiratory metabolism pathways were also significantly altered (Fig. 1D, E). Specifically, most of the glycolytic and pentose phosphate pathway (PPP) enzymes were up-regulated in the resistant cells, whereas the tri-carboxylic acid (TCA) cycle and electron transport chain proteins were down-regulated (Fig. 2). Such a metabolic switch is a characteristic feature of the Warburg effect [23], which is considered a survival mechanism in cancerous tissues [24] that also mediates resistance to apoptosis [25] and to oxidative stress [26]. The underlying mechanisms leading to the Warburg effect are not well understood. However, a number of oncogenes, including c-MYC, are known to enhance the expression of glycolytic enzymes, such as lactate dehydrogenase (LDHA) [27]. Although c-MYC was not quantified by SILAC, its mRNA was 1.8-fold up-regulated in the melphalan-resistant cells (S2 Table) and 1.5-fold upregulated as quantified by western blot (data not shown).

### Melphalan-resistant cells display markedly increased sensitivity towards inhibitors targeting turnover of glycolytic intermediates

**Fig 3:**
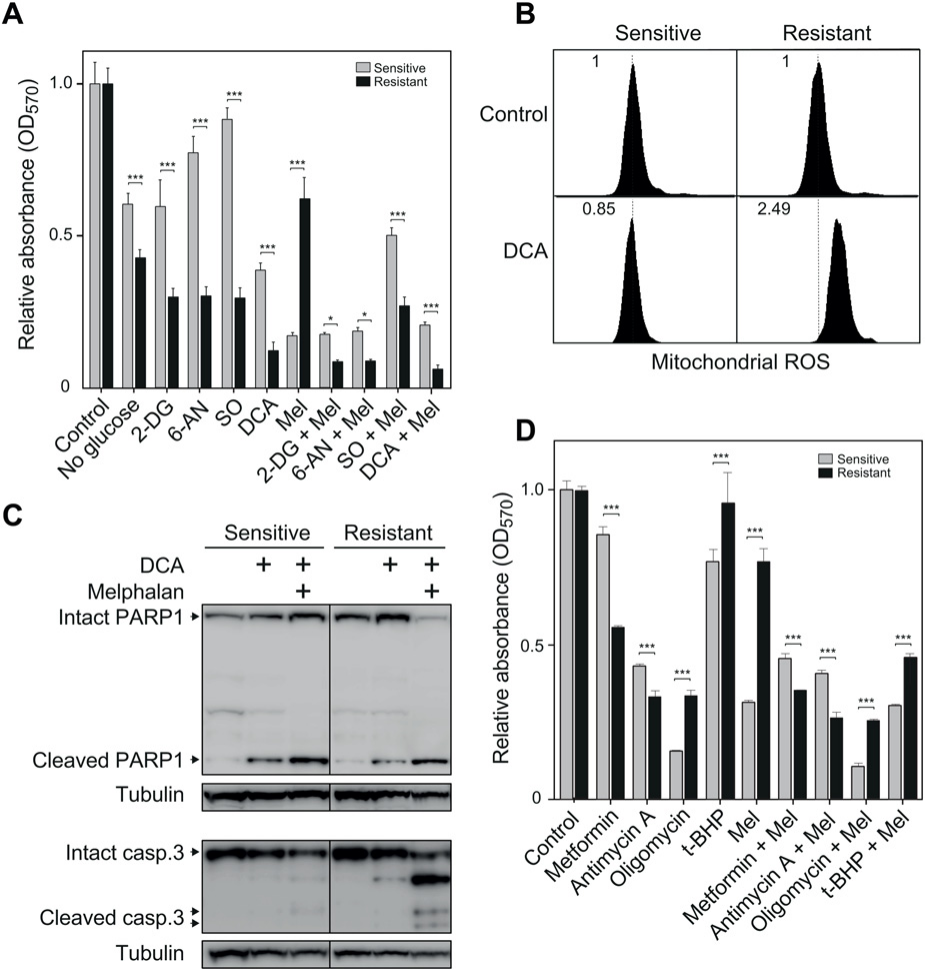
Melphalan-resistant cells are selectively sensitive to inhibitors targeting enzymes in glycolytic- and pentose phosphate pathways as well as complexes in the mitochondrial electron transport chain. (A), Bar diagram illustrating effect of inhibitors targeting various enzymes in carbohydrate metabolism (2-DG, 1 mM, 6-AN, 1 mM, SO, 20 mM, DCA, 10 mM) when administered in the presence or absence of 2.5 μM melphalan (Mel). (B), DCA treatment selectively mediates increased mitochondrial ROS in the resistant cells. (C), Western analysis of the apoptotic markers PARP1 (upper panels) and caspase-3 (bottom panels) illustrating increased cleavage of both proteins subsequent to co-treatment of the resistant cells with DCA and melphalan. (D), Bar diagram illustrating effect of inhibitors targeting the mitochondrial electron transport chain or ATP synthesis (metformin, 3 mM, antimycin A, 30 μM, oligomycin, 10 μM) and the general pro-oxidant tBHP (20 μM) when administered in the presence or absence of 2.5 μM melphalan. (E), Western analysis of AKT1 and pAKT1 in the sensitive and resistant cells.

To further analyze the significance of carbohydrate metabolism in melphalan resistance, sensitive and resistant cells were grown in the presence or absence of glucose. Glucose deprivation had a significantly stronger growth-inhibitory effect in resistant cells compared to sensitive cells (Fig. 3A). We then subjected both cell lines to treatment with enzyme inhibitors that target different steps in glycolysis and the PPP. 2-deoxy-D-glucose (2-DG) inhibits hexokinase (HK) and phosphoglucoisomerase (GPI) and thus funneling of precursors into both glycolysis and PPP. Blocking glycolysis causes ATP depletion, cell cycle arrest and cell death, while blocking PPP inhibits NADPH synthesis and reduces the antioxidant capacity of the cells. 6-aminonicotinamide (6-AN) potently inhibits glucose-6-phosphate dehydrogenase (G6PD) and selectively inhibits PPP. G6PD was more than two-fold up-regulated in the melphalan-resistant cells according to the SILAC analyses. Sodium oxamate (SO) inhibits the conversion of pyruvate to lactate by LDHA, thus inhibiting glycolysis by depleting NAD^+^ [28]. Finally, dichloroacetate (DCA) inhibits all pyruvate dehydrogenase kinase (PDK) isoenzymes, thus increasing the activity of pyruvate dehydrogenase (PDH) and increasing the acetyl-CoA introduced into the TCA cycle [29]. All of these inhibitors had a significantly stronger growth-inhibitory effect upon melphalan-resistant cells and this was also observed when inhibitors were combined with 2.5 μM melphalan (Fig. 3A). This corroborates that a metabolic switch towards aerobic glycolysis contributes to the melphalan-resistant phenotype.

### Melphalan-resistant cells display increased tolerance to overall oxidative stress, but are sensitive to mitochondrial electron transport inhibitors

DCA mediated the largest selective effect against the resistant cells (Fig. 3A). This inhibitor does not directly target glycolytic or PPP enzymes. We thus measured mitochondrial ROS subsequent to DCA-treatment to examine whether increased metabolic flux into mitochondria was less tolerated by the melphalan-resistant cells. As illustrated in Fig. 3B increased mitochondrial ROS was selectively induced in the melphalan-resistant cells subsequent to DCA-treatment. Western analysis of PARP1 and caspase-3 revealed weak induction of PARP1 cleavage in both cell lines subsequent to DCA treatment, whereas combined DCA/melphalan treatment mediated markedly increased cleavage of both PARP1 and caspase-3 in the melphalan-resistant cells (Fig. 3C).

Increased mitochondrial stress may reduce the mitochondrial capability to regenerate NAD^+^ to support glycolytic ATP-production. This would more severely impact the resistant cells given their increased dependence upon glycolytic ATP-production. We tested this by treating the cells with electron transport chain inhibitors (metformin and antimycin A) or with the ATP synthase inhibitor oligomycin. Metformin and antimycin A were more toxic to the resistant cells both in the presence and absence of 2.5 μM melphalan, whereas oligomycin was more toxic to the sensitive cells under both conditions (Fig. 3D). Oligomycin inhibits mitochondrial ATP synthase and does not directly affect the NAD^+^-generating steps. These results further confirm the increased dependence of the melphalan-resistant cells to glycolytic ATP production.

The melphalan-resistant cells were also more tolerant towards the unspecific pro-oxidant tert-butyl hydroperoxide (t-BHP) (Fig. 3D). The cytotoxic effects of t-BHP have been shown to be counteracted by activation of the NRF2-mediated stress response [30]. In affirmation of this, we observed that NRF2 mRNA was significantly up-regulated (1.5 ×) in the resistant cells and the NRF2-mediated oxidative stress response was reported to be significantly (p = 1.03 × 10^-4^) modulated in the IPA analysis. From these results we concluded that the melphalan-resistant cells are more tolerant of oxidative stress, mediated at least partially by NRF2, but are selectively sensitive towards drugs that inhibit NAD^+^ regeneration.

### Lactate accumulation and IL8/VEGF signaling contribute to increased survival in the melphalan-resistant cells

**Fig 4:**
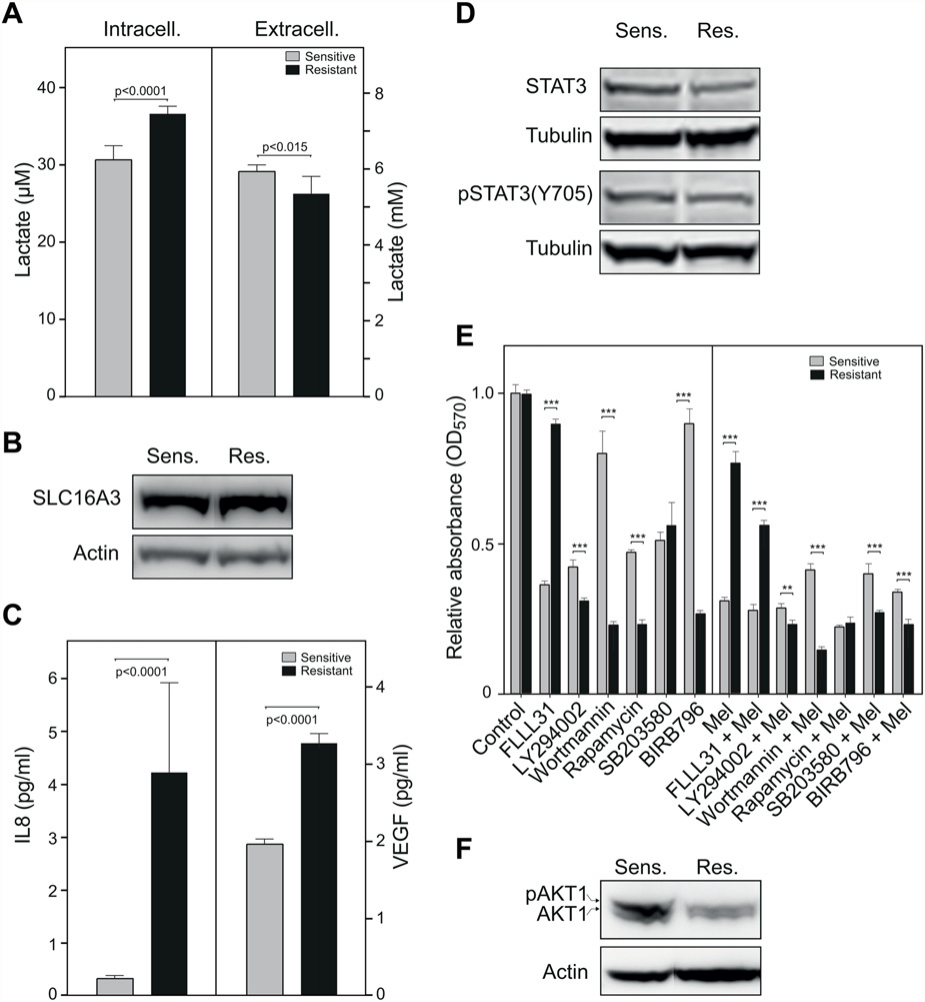
Lactate accumulates in melphalan resistant cells and may mediate increased IL8- and VEGF signaling. (A), Bar diagrams demonstrating significantly increased lactate accumulation in melphalan-resistant cells (left panel), and significantly lower lactate in the extracellular medium (right panel). (B), Western analysis revealed no significant change in expression of the major lactate exporter SLC16A3 (C), Bioplex cytokine profiling demonstrated markedly elevated synthesis of IL8 (left panel) and VEGF (right panel) in the melphalan-resistant cells. (D), Western analysis demonstrated no induced activation of STAT3 in the melphalan resistant cells as probed with anti-pSTAT3 (Y705 antibody). (E), Bar diagram illustrating the effects of various inhibitors targeting STAT3 (FLLL31, 3μM), PI3-kinases (LY294002, 10 μM, wortmannin, 2 μM, rapamycin, 5nM) and p38 MAP kinases (SB203580, 10 μM, BIRB796, 10 μM). (F), Western analysis demonstrating down-regulation of p-AKT1 and AKT1 in the melphalan-resistant cells.

The observed metabolic shift should result in increased lactate production in the resistant cells to regenerate NAD^+^ and facilitate glycolytic ATP production. We found a higher intracellular but lower extracellular lactate content in the melphalan-resistant cells (Fig. 4A), indicating that lactate is retained in the melphalan-resistant cells. Lactate is exported from cells by the monocarboxylate transporters of the SLC16 family, of which SLC16A3 (MCT4) is the major lactate transporter in leukocytes [31]. SLC16A3 was 4.7-fold down-regulated at the mRNA level in the melphalan-resistant cells (S2 Table), but was not quantified in the SILAC analysis. Western analysis of SLC16A3 revealed no significant reduction in the melphalan-resistant cells (Fig. 4B).

Lactate can serve as metabolic fuel in certain cell types via the proposed cytoplasmic/mitochondrial lactate shuttle [32,33]. In addition, lactate may act as a signaling molecule inducing a pseudo-hypoxic condition leading to up-regulation of NF-κB/IL-8 and pro-angiogenic VEGF [34]. The latter is also supported by the co-regulated expression of lactate dehydrogenase and VEGF in tumor cells [35]. We found that IL-8 was 4.4-fold up-regulated at the mRNA level (S2 Table) and both IL-8 and VEGF secretion were increased (>12- and 1.7-fold, respectively) in the melphalan-resistant cells (Fig. 4C). IL-8 and VEGF can mediate pro-survival and proliferation responses through alternative downstream pathways. One of the downstream effectors of IL-8 is STAT3 [36], which has emerged as a potential oncogene target in many solid and hematologic cancers, including MM [37]. However, we found no difference in STAT3 mRNA levels in the sensitive and resistant cells (S2 Table). Moreover, western analysis revealed no induction of total- or phosphorylated (pY705) STAT3 protein in the resistant cells (Fig. 4D). We also subjected the cells to treatment with the STAT3 inhibitor FLLL31, and found that the inhibitor selectively reduced viability in the melphalan-sensitive cells both in the absence and presence of 2.5 μM melphalan (Fig. 4E). An explanation to this could be that alternative survival pathways are up-regulated in the melphalan-resistant cells making them less dependent on STAT3 signaling.

To further investigate the impact of IL-8/VEGF signaling, we subjected the cells to inhibitors that target downstream effectors of IL-8 and VEGF, specifically within the PI3K and p38 MAPK signaling pathways. LY294002 and wortmannin are kinase inhibitors that target PI3K, and rapamycin inhibits mTOR downstream of PI3K. All the PI3K/mTOR inhibitors were more cytotoxic to the melphalan-resistant cells and abolished the selective proliferation advantage of the resistant cells observed with 2.5 μM melphalan alone (Fig. 4E). The mTOR pathway has been suggested to be a potential therapeutic target in myeloma characterized by up-regulated AKT1 [38]. We did not observe a difference in mTOR and AKT1 mRNA levels between melphalan-resistant and sensitive cells (S2 Table) and could not quantify the proteins by SILAC. We therefore measured AKT1 by western blot and found that AKT1 and pAKT1 expression is decreased in melphalan-resistant cells (Fig. 4F). Thus, mTOR inhibition does not fully explain the selective cytotoxicity of these inhibitors in melphalan-resistant cells.

The p38 MAPK inhibitor SB203580 is strictly selective for p38α and β, whereas BIRB0796 inhibits p38α, β, δ and γ [39]. In addition, SB203580 inhibits CK1, GSK3, RIP2 and GAK, and BIRB0796 inhibits JNK2 [38]. BIRB796 was much more cytotoxic in the melphalan-resistant cells, having little effect on the resistant cells, whereas SB203580 showed no selectivity towards either cell line when administered alone. However, both inhibitors reversed the selective growth advantage of the melphalan-resistant cells in 2.5 μM melphalan (Fig. 4B). Taken together, the selective cytotoxicity of inhibitors acting downstream of IL-8/VEGF supports a role of IL-8/VEGF-signaling in acquired melphalan resistance in MM.

### A potential role of aldo-keto reductases in melphalan resistance

**Fig 5:**
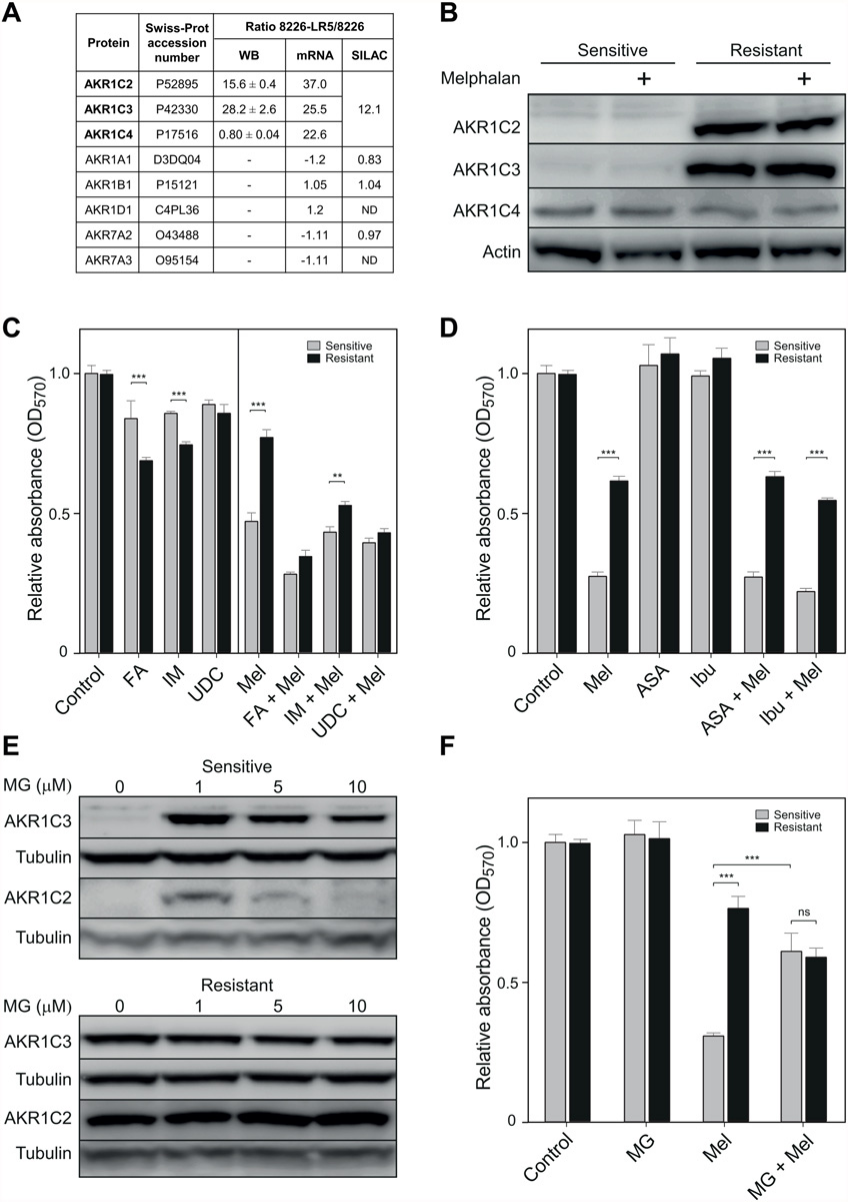
Aldo-keto reductases contribute to melphalan resistance in MM cells. (A), Diagram showing markedly up-regulated levels of the AKR1C family in both SILAC and mRNA analyses, whereas members of other AKR subfamilies are not significantly altered. (B), Western analysis confirmed up-regulation of AKR1C2 and C3, but not C4. (C), Bar diagram illustrating the effects of AKR inhibitors flufenamic acid (FA, 70 μM), ursodeoxycholate (UDC, 16 μM) and indomethacin (IM, 16 μM) when administered in the presence or absence of 2.5 μM melphalan. (D), Bar diagram illustrating that co-treatment of the myeloma cells with NSAIDs acetylsalisylic acid (ASA) or ibuprofen (Ibu) with melphalan does not reverse the melphalan-resistant phenotype. (E), Western analysis demonstrating strong induction of AKR1C3 and a weaker induction of AKR1C2 in the melphalan-sensitive cells (upper panel) subsequent to 20 h incubation in various concentrations of methyl glyoxal (MG). MG treatment did not affect the AKR1C levels in the resistant cells (lower panel). (F), Bar diagram illustrating that that MG pretreatment (1μM) induced a melphalan-resistant phenotype in the parental RPMI8226 cells similar to that of the resistant LR5 cells.

The aldo-keto reductase AKR1C enzymes constitute a family of oxidoreductases that catalyze NADPH-dependent reduction of a wide variety of substrates [40]. Here, AKR1C1–3 regulate ligand occupancy and *trans*-activation of androgen-, estrogen- and progesterone receptors, the peroxisome proliferator-activated receptor (PPARγ) as well as the prostaglandin metabolism [41]. AKRC1 members displayed the most pronounced up-regulated expression in both the SILAC and mRNA profiling analyses (Fig. 5A). Due to their extensive sequence homology (84% to 98%), AKR1C1–4 were collectively reported as 12-fold up-regulated in SILAC. AKR1C2, 3 and 4 mRNA were 37-, 25- and 22-fold up-regulated, respectively. We also measured AKR1C2, C3 and C4 by western blot and observed the most prominent up-regulation of AKR1C2 and C3 (15.6- and 28.2-fold, respectively, Fig. 5A, B). Moreover, enzyme levels did not increase in either cell line after 50 μM melphalan treatment (Fig. 5B), indicating that their up-regulation is a result of adaptation to prolonged treatment with low dose melphalan rather than an immediate stress response mediated by short time exposure to high dose melphalan.

To explore the potential role of AKR1C2 and C3 in the melphalan resistance mechanism, melphalan-sensitive and-resistant cells were treated with aldo-keto reductase inhibitors. Flufenamic acid (FA) is a non-steroidal anti-inflammatory drug (NSAID) that has anti-inflammatory and antipyretic properties and acts as a broad-spectrum AKR1C inhibitor. In addition it modulates several channel activities and is an activator of AMP-activated protein kinase [42]. Indomethacin (IM) is an NSAID commonly used to reduce fever and pain by inhibiting the production of prostaglandins, and inhibits AKR1C3 much more potently than AKRC1 and AKRC2 [43,44]. IM and FA are also nanomolar and micromolar inhibitors, respectively, of COX-1/2 (PTGS1/2) [45]. Previous work suggests, however, that the antiproliferative effect of IM upon myeloma cells is independent of COX-2 [46]. Notably FA and IM were moderately selectively cytotoxic to the melphalan-resistant cells when administered alone (Fig. 5C, left panel). When administered together with melphalan, both inhibitors markedly reversed the resistant phenotype (Fig. 5C, right panel). To further investigate the specific role of AKR1C in the melphalan-resistant cells we also tested the non-NSAID drug ursodeoxycholate (UDC). UDC is a bile acid drug that reduces cholesterol absorption and is used to treat primary biliary cirrhosis. It inhibits both AKR1C2 and C3 and has been shown to have pro- and anti-apoptotic properties depending on the cell lines and experimental systems employed [47,48]. Importantly, UDC mediated a nearly complete reversal of the melphalan-resistant phenotype, similar to that seen with FA (Fig. 5C, right panel). These results strongly suggest that AKR1C2 and C3 contribute to melphalan resistance in the myeloma cells. However, to further investigate whether a general effect mediated by NSAID treatment was involved in reversal of resistance, the cells were also treated with acetylsalicylic acid or ibuprofen. As demonstrated in Fig. 5D, such treatment did not mediate any change in the relative resistance of the myeloma cells, further substantiating that FA and IM primarily affect melphalan resistance by AKR1C inhibition.

In a recent study of aldehyde toxicity in neuroblastoma cells, Lyon et al. [49] demonstrated that the AKR1C3 protein level could be induced by short-time treatment with sub-lethal doses of either methyl glyoxal or 4-hydroxy-2-nonenal (4HNE). These reactive aldehydes are increased in several neurodegenerative diseases [50] and recombinant AKR1C1–3 have been shown to possess high specific activities against 4HNE [51]. Incubation of the melphalan-sensitive and-resistant cells in 1 μM methyl glyoxal for 24 h mediated a strong up-regulation of AKR1C3 in the melphalan-sensitive cells whereas the AKR1C3 level remained unaltered in the resistant cells. AKR1C2 was also up-regulated by the pretreatment, but much weaker than AKR1C3 (Fig. 5E). The treatment did not mediate any cytotoxic effect in the cells (Fig. 5F). However, subsequent treatment of the cells in 2.5 μM melphalan demonstrated that the methyl glyoxal pretreatment mediated a resistant phenotype in the parental RPMI8226 cells resembling that of the LR5 derivative (Fig. 5F). These results corroborate the inhibitor studies and support a role of AKR1C aldo-keto reductases in mediating melphalan resistance in the myeloma cells.

## Discussion

MM is a complex and heterogeneous disease and as such, many factors likely contribute to acquired melphalan resistance. Here we used a transcriptomic and proteomic approach to identify key proteins involved in melphalan resistance in isogenic myeloma cell lines. We thereby demonstrated an active involvement of a metabolic switch in melphalan-resistant cells towards aerobic glycolysis accompanied by an increased tolerance towards oxidative stress. Aerobic glycolysis (Warburg effect) has been largely ignored in cytostatic resistance research, but several recent findings indicate that combining cytostatics with targeted inhibition of aberrant cellular metabolism may overcome chemorefractoriness [52]. We similarly targeted the glycolytic pathway with 2-DG, 6-AN and SO and found that they were selectively cytotoxic to melphalan-resistant cells. 2-DG has recently been implemented in phase-I clinical trials in patients with advanced solid tumors [53] and its cytotoxicity has been partially attributed to glycolytic inhibition.

DCA was also selectively cytotoxic to the melphalan-resistant cells, and this is corroborated by Sanchez *et al*., who demonstrated a selective effect of DCA in multiple myeloma cell lines having high aerobic glycolysis [54]. DCA also increased the sensitivity of these cells towards the proteasome inhibitor bortezomib. ROS induction has been demonstrated to play a critical role in bortezomib-induced apoptosis by disrupting the mitochondrial membrane potential [55], and DCA has been shown to synergistically inhibit proliferation in melanoma cells with induced mitochondrial ROS [56]. Melphalan and other DNA cross-linkers increase mitochondrial ROS in HeLa and HCT cells [57] and oxidative stress has been shown to increase melphalan cytotoxicity in chronic myeloid leukemia cells [58]. Thus, DCA could potentiate the effects of melphalan (as well as other cytostatic drugs) by inducing cellular oxidative stress, possibly because the mitochondrial metabolism is down-regulated to counterbalance the oxidative stress induced by continuous drug exposure.

In addition to mediating increased mitochondrial ROS, DCA also depletes substrate from the NAD^+^-generating LDHA step. This slows glycolytic ATP production, rendering the melphalan-resistant cells more sensitive to treatment. The increased reliance of NAD^+^-dependent aerobic glycolysis of the melphalan-resistant cells is also supported by the selective effect of the electron transport chain inhibitors metformin and antimycin A, which target the NAD^+^-generating steps. Metformin is well tolerated and widely used in the treatment of diabetes, and it has been shown to suppress breast and pancreatic tumor development and reprogram ovarian and breast cancer cells to a non-cancerous state [59–63]; however, the molecular mechanisms by which metformin displays an anti-cancer effect and is selectively cytotoxic to melphalan-resistant myeloma cells require further study.

Both IL-8 and VEGF are important mediators of hypoxia and oxidative stress signaling that stimulate proliferation and survival of MM cells [64]. In addition, IL-8 is a potent pro-inflammatory cytokine. Downstream signaling involves complex kinase networks including MAPKs, PI3Ks and PKC [36]. The two PI3K inhibitors LY294002 and wortmannin were selectively cytotoxic to the melphalan-resistant cells. LY294002 broadly targets PI3Ks and has been shown to inhibit CK2, mTOR and GSK3B [65], while wortmannin targets PI3K-C2β and weakly inhibits mTOR and DNA-PK [66,67]. The highly specific mTOR-inhibitor rapamycin was also selectively cytotoxic to the resistant cells. Rapamycin inhibits mTORC1 and may also inhibit mTORC2 in some cell types [68]. In the melphalan-resistant cells, mTOR signaling is apparently not AKT dependent, since AKT1- and pAKT1-levels were both reduced. Moreover, PTEN, which negatively regulates AKT, was more than twofold up-regulated at the mRNA level in the resistant cells. AKT1-independent activation of mTOR has been reported in several cell types, including B-lymphocyte cell lines [69] and follicular lymphoma cells [70]. In the tumor syndrome tuberous sclerosis (TSC) mTOR is indirectly activated by MAPK-mediated phosphorylation of TSC1 and 2 that alleviates inhibition of mTOR [71]. Notably we find that both TSC1 and 2 are significantly down-regulated at the mRNA level in the resistant cells (S2 Table), potentially contributing to increased functional mTOR. Recent studies have assigned a central role of mTOR2C in the control of metabolic reprogramming in cancer cells. This function is AKT-independent and rather involves up-regulation of c-MYC [72,73], which was found to be 1.8-fold up-regulated at the mRNA level in the resistant cells. In addition, the glucocorticoid-stimulated kinase SGK1, which is a downstream target of mTORC2 and an important mediator of AKT-independent mTOR signaling [74] was fourfold up-regulated at the mRNA level in the melphalan-resistant cells (S2 Table).

The p38MAPK inhibitors SB203580 and BIRB796 were both differently cytotoxic to the cell lines. These inhibitors also exhibit different specificities, with SB230580 inhibiting p38α and β, as well as CK1, GSK3, RIP2 and GAK, while BIRB796 inhibits p38α, β, γ and δ as well as JNK2. The overlapping specificities of the inhibitors as well as the inherent complexity and crosstalk between the various signaling pathways renders elucidation of the exact mechanisms involved to be extremely challenging; however, the selective cytotoxicity of BIRB796 towards the melphalan-resistant cells warrant further investigation into the involvement of JNK2 in melphalan resistance. JNK2 has a central role in stress signaling, including oxidative stress, and also mediates secretion of pro-inflammatory IL-8, thus potentially forming an autocrine stimulatory loop in the melphalan-resistant cells.

The involvement of AKR1C enzymes in melphalan resistance was substantiated by inhibitor studies as well as the ability of mehyl glyoxal-induced AKR1C3 to induce melphalan-resistance in the parental RPMI8226 cells. The AKR1C subfamily plays essential roles in the metabolism of steroid hormones and bile acids, and is involved in the pre-receptor regulation of nuclear and membrane-bound receptors [75]. Dysregulated AKR1C2 and C3 expression has been linked to the development of prostate, breast and endometrial carcinomas as well as leukemias, likely due to their ability to modify steroid hormones and prostaglandins (PDGs) [75]. Moreover, AKR1C3 has been identified as a potential therapeutic target in leukemia because its PGD2 reductase activity may prevent cell differentiation [76]. This activity also prevents formation of the pro-apoptotic 15-deoxy-PGJ2 [77]. In a recent drug screen to identify specific AKR1C3 inhibitors, tetracycline was initially identified as a potential AKR1C3-selective inhibitor. However subsequent analysis by mass spectrometry and NMR revealed that the active inhibitor was a breakdown product of tetracycline, (4-methyl(de-dimethylamine)-tetracycline (4-MDDT). Treatment of leukemia cells with 4-MDDT did not recapitulate the anti-leukemic property of pan-AKR1C inhibitors, suggesting that multiple AKR1C inhibition is likely required to elicit an anticancer effect in leukemia [78], in agreement with our findings. In addition to endogenous substrates, AKR1C enzymes have been implicated in the metabolism of several exogenous substrates, including drugs, carcinogens and reactive aldehydes such as 4-hydroxynonenal (4HNE) [49]. AKR1C1–3 has been shown to possess a high specific activity for 4HNE reduction [51], one of the most cytotoxic products of lipid peroxidation that is likely formed by melphalan-induced cellular peroxide [79] and increased plasma lipid peroxidation. A potential role of AKR1C1–3 in melphalan resistance could thus be a result of 4HNE processing. Although currently speculative, the AKR1C enzymes might also have a more subtle role in regulating the redox balance of cells under oxidative stress. They act primarily as reductases *in vivo*, (*K*_*d*_ for NADPH in the nanomolar range) and could potentially contribute to influence the NADPH/NADP^+^ balance. Since NADPH potently inhibits NAD^+^-dependent oxidation reactions at low μM concentrations, such regulation could ensure sufficient NAD^+^ to allow active glycolytic ATP-generation in the melphalan-resistant cells.

## Conclusions

Our results reveal the presence of several previously unrecognized mechanisms for acquired drug resistance against melphalan. The SILAC profiling approach identified a switch in metabolism towards aerobic glycolysis as well as modulated oxidative stress signaling in the resistant cells. The metabolic switch was not evident from the mRNA results alone and underscores the power of quantitative proteomic profiling to identify alterations in biological pathways under significant translational control, such as metabolic pathways during chronic stress [80]. The present in-depth analysis of a melphalan-sensitive cell line and its resistant derivative highlights several promising targets within metabolic and oxidative stress response pathways that should be further explored to overcome melphalan resistance in MM. Such studies are now in progress in our laboratory.

## Supporting Information

S1 Table Expression levels of proteins in melphalan-resistant and-sensitive RPMI8226 cells as quantified by SILAC. (TXT)

S2 Table Differentially expressed mRNAs in melphalan-resistant and-sensitive RPMI8226 cells as determined from Illumina analysis. (CSV)

## References

[1] Raab MS, Podar K, Breitkreutz I, Richardson PG, Anderson KC (2009) Multiple myeloma. Lancet 374;9686: 324–339. 10.1016/S0140-6736(09)60221-X 19541364

[2] Lonial S, Cavenagh J (2009) Emerging combination treatment strategies containing novel agents in newly diagnosed multiple myeloma. Br J Haematol 145;6: 681–708. 10.1111/j.1365-2141.2009.07649.x 19344388

[3] Kyle RA, Rajkumar SV (2009) Treatment of multiple myeloma: a comprehensive review. Clin Lymphoma Myeloma 9;4: 278–288. 10.3816/CLM.2009.n.056 19717377 PMC3910142

[4] Povirk LF, Shuker DE (1994) DNA damage and mutagenesis induced by nitrogen mustards. Mutat Res 318;3: 205–226. 7527485 10.1016/0165-1110(94)90015-9

[5] Mohamed D, Linscheid M (2008) Separation and identification of trinucleotide-melphalan adducts from enzymatically digested DNA using HPLC-ESI-MS. Anal Bioanal Chem 392;5: 805–817. 10.1007/s00216-008-2236-0 18622599

[6] Ahmed AE, Hsu TF, el-Azhary RA, Moawad H, Farrish HH Jr, et al (1982) Tissue distribution and macromolecular interactions of 14[C-ring] melphalan in the rat. Cancer Chemother Pharmacol 8;3: 271–276. 7127659 10.1007/BF00254049

[7] Homolya L, Varadi A, Sarkadi B (2003) Multidrug resistance-associated proteins: Export pumps for conjugates with glutathione, glucuronate or sulfate. Biofactors 17;1–4: 103–114. 12897433 10.1002/biof.5520170111

[8] Bellamy WT, Dalton WS, Gleason MC, Grogan TM, Trent JM (1991) Development and characterization of a melphalan-resistant human multiple myeloma cell line. Cancer Res 51;3: 995–1002. 1988143

[9] Rothbarth J, Vahrmeijer AL, Mulder GJ (2002) Modulation of cytostatic efficacy of melphalan by glutathione: mechanisms and efficacy. Chem Biol Interact 140;2: 93–107. 12076518 10.1016/s0009-2797(02)00014-5

[10] Spanswick VJ, Craddock C, Sekhar M, Mahendra P, Shankaranarayana P, et al (2002) Repair of DNA interstrand crosslinks as a mechanism of clinical resistance to melphalan in multiple myeloma. Blood 100;1: 224–229. 12070031 10.1182/blood.v100.1.224

[11] Chen Q, Van der Sluis PC, Boulware D, Hazlehurst LA, Dalton WS (2005) The FA/BRCA pathway is involved in melphalan-induced DNA interstrand cross-link repair and accounts for melphalan resistance in multiple myeloma cells. Blood 106;2: 698–705. 15802532 10.1182/blood-2004-11-4286 PMC1895179

[12] Yarde DN, Oliveira V, Mathews L, Wang X, Villagra A, et al (2009) Targeting the Fanconi anemia/BRCA pathway circumvents drug resistance in multiple myeloma. Cancer Res 69;24: 9367–9375. 10.1158/0008-5472.CAN-09-2616 19934314 PMC4519834

[13] Sousa MM, Zub KA, Aas PA, Hanssen-Bauer A, Demirovic A, et al (2013) An inverse switch in DNA base excision and strand break repair contributes to melphalan resistance in multiple myeloma cells. PLOS ONE 8;2: e55493 10.1371/journal.pone.0055493 23405159 PMC3566207

[14] Xie J, Zhang L, Li M, Du J, Zhou L, et al (2014) Functional analysis of the involvement of apurinic/apyrimidinic endonuclease 1 in the resistance to melphalan in multiple myeloma. BMC Cancer 14;1: 11. 24400589 10.1186/1471-2407-14-11 PMC3900260

[15] Das GC, Bacsi A, Shrivastav M, Hazra TK, Boldogh I (2006) Enhanced gamma-glutamylcysteine synthetase activity decreases drug-induced oxidative stress levels and cytotoxicity. Mol Carcinog 45;9: 635–647. 16491484 10.1002/mc.20184

[16] Oancea M, Mani A, Hussein MA, Almasan A (2004) Apoptosis of multiple myeloma. Int J Hematol 80;3: 224–231. 15540896 10.1532/IJH97.04107 PMC1193518

[17] Gentleman RC, Carey VJ, Bates DM, Bolstad B, Dettling M, et al (2004) Bioconductor: open software development for computational biology and bioinformatics. Genome Biol 5;10: R80 15461798 10.1186/gb-2004-5-10-r80 PMC545600

[18] Smyth GK (2004) Linear models and empirical bayes methods for assessing differential expression in microarray experiments. Stat Appl Genet Mol Biol 3: Article3 16646809 10.2202/1544-6115.1027

[19] NCBI Gene Expression Omnibus. Available: http://www.ncbi.nlm.nih.gov/geo/query/acc.cgi?acc=GSE60970. Accessed 2015 Feb 5.

[20] Shevchenko A, Wilm M, Vorm O, Mann M (1996) Mass spectrometric sequencing of proteins silver-stained polyacrylamide gels. Anal Chem 68;5: 850–858. 8779443 10.1021/ac950914h

[21] Vizcaino JA, Deutsch EW, Wang R, Csordas A, Reisinger F, et al (2014) ProteomeXchange provides globally coordinated proteomics data submission and dissemination. Nat Biotechnol 32;3: 223–226. 10.1038/nbt.2839 24727771 PMC3986813

[22] EMBL-EBI PRIDE Database. Available: http://www.ebi.ac.uk/pride/archive/projects/PXD001276. Accessed 2015 Feb 5.

[23] Warburg O (1956) On the origin of cancer cells. Science 123;3191: 309–314. 13298683 10.1126/science.123.3191.309

[24] Upadhyay M, Samal J, Kandpal M, Singh OV, Vivekanandan P (2013) The Warburg effect: insights from the past decade. Pharmacol Ther 137;3: 318–330. 10.1016/j.pharmthera.2012.11.003 23159371

[25] Plas DR, Thompson CB (2002) Cell metabolism in the regulation of programmed cell death. Trends Endocrinol Metab 13;2: 75–78. 11854022 10.1016/s1043-2760(01)00528-8

[26] DeBerardinis RJ (2008) Is cancer a disease of abnormal cellular metabolism? New angles on an old idea. Genet Med 10;11: 767–777. 10.1097/GIM.0b013e31818b0d9b 18941420 PMC2782690

[27] Dang CV, Le A, Gao P (2009) MYC-induced cancer cell energy metabolism and therapeutic opportunities. Clin Cancer Res 15;21: 6479–6483. 10.1158/1078-0432.CCR-09-0889 19861459 PMC2783410

[28] Wu M, Neilson A, Swift AL, Moran R, Tamagnine J, et al (2007) Multiparameter metabolic analysis reveals a close link between attenuated mitochondrial bioenergetic function and enhanced glycolysis dependency in human tumor cells. Am J Physiol Cell Physiol 292;1: C125–136. 16971499 10.1152/ajpcell.00247.2006

[29] Niewisch MR, Kuci Z, Wolburg H, Sautter M, Krampen L, et al (2012) Influence of dichloroacetate (DCA) on lactate production and oxygen consumption in neuroblastoma cells: is DCA a suitable drug for neuroblastoma therapy? Cell Physiol Biochem 29;3–4: 373–380. 22508045 10.1159/000338492

[30] Dowell JA, Johnson JA (2013) Mechanisms of Nrf2 protection in astrocytes as identified by quantitative proteomics and siRNA screening. PLOS ONE 8;7: e70163 10.1371/journal.pone.0070163 23922950 PMC3726381

[31] Halestrap AP, Meredith D (2004) The SLC16 gene family-from monocarboxylate transporters (MCTs) to aromatic amino acid transporters and beyond. Pflugers Arch 447;5: 619–628. 12739169 10.1007/s00424-003-1067-2

[32] Pizzuto R, Paventi G, Porcile C, Sarnataro D, Daniele A, et al (2012) l-Lactate metabolism in HEP G2 cell mitochondria due to the l-lactate dehydrogenase determines the occurrence of the lactate/pyruvate shuttle and the appearance of oxaloacetate, malate and citrate outside mitochondria. Biochim Biophys Acta 1817;9: 1679–1690. 10.1016/j.bbabio.2012.05.010 22659615

[33] Brooks GA, Dubouchaud H, Brown M, Sicurello JP, Butz CE (1999) Role of mitochondrial lactate dehydrogenase and lactate oxidation in the intracellular lactate shuttle. Proc Natl Acad Sci U S A 96;3: 1129–1134. 9927705 10.1073/pnas.96.3.1129 PMC15362

[34] Vegran F, Boidot R, Michiels C, Sonveaux P, Feron O (2011) Lactate influx through the endothelial cell monocarboxylate transporter MCT1 supports an NF-kappaB/IL-8 pathway that drives tumor angiogenesis. Cancer Res 71;7: 2550–2560. 10.1158/0008-5472.CAN-10-2828 21300765

[35] Kim HS, Lee HE, Yang HK, Kim WH (2013) High Lactate Dehydrogenase 5 Expression Correlates with High Tumoral and Stromal Vascular Endothelial Growth Factor Expression in Gastric Cancer. Pathobiology 81;2: 78–85. 10.1159/000357017 24401755

[36] Waugh DJ, Wilson C (2008) The interleukin-8 pathway in cancer. Clin Cancer Res 14;21: 6735–6741. 10.1158/1078-0432.CCR-07-4843 18980965

[37] Page BD, Croucher DC, Li ZH, Haftchenary S, Jimenez-Zepeda VH, et al (2013) Inhibiting aberrant signal transducer and activator of transcription protein activation with tetrapodal, small molecule Src homology 2 domain binders: promising agents against multiple myeloma. J Med Chem 56;18: 7190–7200. 10.1021/jm3017255 23968501

[38] Shi Y, Yan H, Frost P, Gera J, Lichtenstein A (2005) Mammalian target of rapamycin inhibitors activate the AKT kinase in multiple myeloma cells by up-regulating the insulin-like growth factor receptor/insulin receptor substrate-1/phosphatidylinositol 3-kinase cascade. Mol Cancer Ther 4;10: 1533–1540. 16227402 10.1158/1535-7163.MCT-05-0068

[39] Kuma Y, Sabio G, Bain J, Shpiro N, Marquez R, et al (2005) BIRB796 inhibits all p38 MAPK isoforms in vitro and in vivo. J Biol Chem 280;20: 19472–19479. 15755732 10.1074/jbc.M414221200

[40] Penning TM (2014) The aldo-keto reductases (AKRs): Overview. Chem Biol Interact. pii: S0009-2797(14)00273–7. 10.1016/j.cbi.2014.09.024 PMC4388799 25304492

[41] Penning TM, Drury JE (2007) Human aldo-keto reductases: Function, gene regulation, and single nucleotide polymorphisms. Arch Biochem Biophys 464;2: 241–250. 17537398 10.1016/j.abb.2007.04.024 PMC2025677

[42] Chi Y, Li K, Yan Q, Koizumi S, Shi L, et al (2011) Nonsteroidal anti-inflammatory drug flufenamic acid is a potent activator of AMP-activated protein kinase. J Pharmacol Exp Ther 339;1: 257–266. 10.1124/jpet.111.183020 21765041

[43] Byrns MC, Penning TM (2009) Type 5 17beta-hydroxysteroid dehydrogenase/prostaglandin F synthase (AKR1C3): role in breast cancer and inhibition by non-steroidal anti-inflammatory drug analogs. Chem Biol Interact 178:1–3: 221–227. 19010312 10.1016/j.cbi.2008.10.024 PMC3076957

[44] Bauman DR, Rudnick SI, Szewczuk LM, Jin Y, Gopishetty S, et al (2005) Development of nonsteroidal anti-inflammatory drug analogs and steroid carboxylates selective for human aldo-keto reductase isoforms: potential antineoplastic agents that work independently of cyclooxygenase isozymes. Mol Pharmacol 67;1: 60–68. 15475569 10.1124/mol.104.006569

[45] Ouellet M, Falgueyret JP, Percival MD (2004) Detergents profoundly affect inhibitor potencies against both cyclo-oxygenase isoforms. Biochem J 377;Pt3: 675–684. 14510637 10.1042/BJ20030969 PMC1223887

[46] Ding J, Tsuboi K, Hoshikawa H, Goto R, Mori N, et al (2006) Cyclooxygenase isozymes are expressed in human myeloma cells but not involved in anti-proliferative effect of cyclooxygenase inhibitors. Mol Carcinog 45;4: 250–259. 16385588 10.1002/mc.20175

[47] Saeki T, Yui S, Hirai T, Fujii T, Okada S, et al (2012) Ursodeoxycholic acid protects colon cancer HCT116 cells from deoxycholic acid-induced apoptosis by inhibiting apoptosome formation. Nutr Cancer 64;4: 617–626. 10.1080/01635581.2012.669876 22497644

[48] Ikegami T, Matsuzaki Y, Al Rashid M, Ceryak S, Zhang Y, et al (2006) Enhancement of DNA topoisomerase I inhibitor-induced apoptosis by ursodeoxycholic acid. Mol Cancer Ther 5;1: 68–79. 16432164 10.1158/1535-7163.MCT-05-0107

[49] Lyon RC, Li D, McGarvie G, Ellis EM (2013) Aldo-keto reductases mediate constitutive and inducible protection against aldehyde toxicity in human neuroblastoma SH-SY5Y cells. Neurochem Int 62;1: 113–121. 10.1016/j.neuint.2012.10.007 23084985

[50] Reed TT (2011) Lipid peroxidation and neurodegenerative disease. Free Radic Biol Med 51;7: 1302–1319. 10.1016/j.freeradbiomed.2011.06.027 21782935

[51] Burczynski ME, Sridhar GR, Palackal NT, Penning TM (2001) The reactive oxygen species—and Michael acceptor-inducible human aldo-keto reductase AKR1C1 reduces the alpha,beta-unsaturated aldehyde 4-hydroxy-2-nonenal to 1,4-dihydroxy-2-nonene. J Biol Chem 276;4: 2890–2897. 11060293 10.1074/jbc.M006655200

[52] Zhao Y, Butler EB, Tan M (2013) Targeting cellular metabolism to improve cancer therapeutics. Cell Death Dis 4: e532 10.1038/cddis.2013.60 23470539 PMC3613838

[53] Raez LE, Papadopoulos K, Ricart AD, Chiorean EG, Dipaola RS, et al (2013) A phase I dose-escalation trial of 2-deoxy-D-glucose alone or combined with docetaxel in patients with advanced solid tumors. Cancer Chemother Pharmacol 71;2: 523–530. 10.1007/s00280-012-2045-1 23228990

[54] Sanchez WY, McGee SL, Connor T, Mottram B, Wilkinson A, et al (2013) Dichloroacetate inhibits aerobic glycolysis in multiple myeloma cells and increases sensitivity to bortezomib. Br J Cancer 108;8: 1624–1633. 10.1038/bjc.2013.120 23531700 PMC3668464

[55] Ling YH, Liebes L, Zou Y, Perez-Soler R (2003) Reactive oxygen species generation and mitochondrial dysfunction in the apoptotic response to Bortezomib, a novel proteasome inhibitor, in human H460 non-small cell lung cancer cells. J Biol Chem 278;36: 33714–33723. 12821677 10.1074/jbc.M302559200

[56] Kluza J, Corazao-Rozas P, Touil Y, Jendoubi M, Maire C, et al (2012) Inactivation of the HIF-1alpha/PDK3 signaling axis drives melanoma toward mitochondrial oxidative metabolism and potentiates the therapeutic activity of pro-oxidants. Cancer Res 72;19: 5035–5047. 10.1158/0008-5472.CAN-12-0979 22865452

[57] Sharaf el dein O, Gallerne C, Brenner C, Lemaire C (2012) Increased expression of VDAC1 sensitizes carcinoma cells to apoptosis induced by DNA cross-linking agents. Biochem Pharmacol 83;9: 1172–1182. 10.1016/j.bcp.2012.01.017 22285227

[58] Verrax J, Dejeans N, Sid B, Glorieux C, Calderon PB (2011) Intracellular ATP levels determine cell death fate of cancer cells exposed to both standard and redox chemotherapeutic agents. Biochem Pharmacol 82;11: 1540–1548. 10.1016/j.bcp.2011.07.102 21843513

[59] Decensi A, Puntoni M, Goodwin P, Cazzaniga M, Gennari A, et al (2010) Metformin and cancer risk in diabetic patients: a systematic review and meta-analysis. Cancer Prev Res (Phila) 3;11: 1451–1461. 10.1158/1940-6207.CAPR-10-0157 20947488

[60] Currie CJ, Poole CD, Jenkins-Jones S, Gale EA, Johnson JA, et al (2012) Mortality after incident cancer in people with and without type 2 diabetes: impact of metformin on survival. Diabetes Care 35;2: 299–304. 10.2337/dc11-1313 22266734 PMC3263862

[61] Anisimov VN, Berstein LM, Egormin PA, Piskunova TS, Popovich IG, et al (2005) Effect of metformin on life span and on the development of spontaneous mammary tumors in HER-2/neu transgenic mice. Exp Gerontol 40;8–9: 685–693. 16125352 10.1016/j.exger.2005.07.007

[62] Schneider MB, Matsuzaki H, Haorah J, Ulrich A, Standop J, et al (2001) Prevention of pancreatic cancer induction in hamsters by metformin. Gastroenterology 120;5: 1263–1270. 11266389 10.1053/gast.2001.23258

[63] Hu T, Chung YM, Guan M, Ma M, Ma J, et al (2014) Reprogramming ovarian and breast cancer cells into non-cancerous cells by low-dose metformin or SN-38 through FOXO3 activation. Sci Rep 4: 5810 10.1038/srep05810 25056111 PMC4108946

[64] Podar K, Anderson KC (2005) The pathophysiologic role of VEGF in hematologic malignancies: therapeutic implications. Blood 105;4: 1383–1395. 15471951 10.1182/blood-2004-07-2909

[65] Gharbi SI, Zvelebil MJ, Shuttleworth SJ, Hancox T, Saghir N, et al (2007) Exploring the specificity of the PI3K family inhibitor LY294002. Biochem J 404;1: 15–21. 17302559 10.1042/BJ20061489 PMC1868829

[66] Hartley KO, Gell D, Smith GC, Zhang H, Divecha N, et al (1995) DNA-dependent protein kinase catalytic subunit: a relative of phosphatidylinositol 3-kinase and the ataxia telangiectasia gene product. Cell 82;5: 849–856. 7671312 10.1016/0092-8674(95)90482-4

[67] Brunn GJ, Hudson CC, Sekulic A, Williams JM, Hosoi H, et al (1997) Phosphorylation of the translational repressor PHAS-I by the mammalian target of rapamycin. Science 277;5322: 99–101. 9204908 10.1126/science.277.5322.99

[68] Sarbassov DD, Ali SM, Sengupta S, Sheen JH, Hsu PP, et al (2006) Prolonged rapamycin treatment inhibits mTORC2 assembly and Akt/PKB. Mol Cell 22;2: 159–168. 16603397 10.1016/j.molcel.2006.03.029

[69] Wlodarski P, Kasprzycka M, Liu X, Marzec M, Robertson ES, et al (2005) Activation of mammalian target of rapamycin in transformed B lymphocytes is nutrient dependent but independent of Akt, mitogen-activated protein kinase/extracellular signal-regulated kinase kinase, insulin growth factor-I, and serum. Cancer Res 65;17: 7800–7808. 16140948 10.1158/0008-5472.CAN-04-4180

[70] Leseux L, Hamdi SM, Al Saati T, Capilla F, Recher C, et al (2006) Syk-dependent mTOR activation in follicular lymphoma cells. Blood 108;13: 4156–4162. 16912221 10.1182/blood-2006-05-026203

[71] Ma L, Chen Z, Erdjument-Bromage H, Tempst P, Pandolfi PP (2005) Phosphorylation and functional inactivation of TSC2 by Erk implications for tuberous sclerosis and cancer pathogenesis. Cell 121;2: 179–193. 15851026 10.1016/j.cell.2005.02.031

[72] Masui K, Tanaka K, Akhavan D, Babic I, Gini B, et al (2013) mTOR complex 2 controls glycolytic metabolism in glioblastoma through FoxO acetylation and upregulation of c-Myc. Cell Metab 18;5: 726–739. 10.1016/j.cmet.2013.09.013 24140020 PMC3840163

[73] Masui K, Cavenee WK, Mischel PS (2014) mTORC2 in the center of cancer metabolic reprogramming. Trends Endocrinol Metab 25: 364–373. 10.1016/j.tem.2014.04.002 24856037 PMC4077930

[74] Mori S, Nada S, Kimura H, Tajima S, Takahashi Y, et al (2014) The mTOR pathway controls cell proliferation by regulating the FoxO3a transcription factor via SGK1 kinase. PLOS ONE 9;2: e88891 10.1371/journal.pone.0088891 24558442 PMC3928304

[75] Rizner TL, Penning TM (2014) Role of aldo-keto reductase family 1 (AKR1) enzymes in human steroid metabolism. Steroids 79: 49–63. 10.1016/j.steroids.2013.10.012 24189185 PMC3870468

[76] Birtwistle J, Hayden RE, Khanim FL, Green RM, Pearce C, et al (2009) The aldo-keto reductase AKR1C3 contributes to 7,12-dimethylbenz(a)anthracene-3,4-dihydrodiol mediated oxidative DNA damage in myeloid cells: implications for leukemogenesis. Mutat Res 662;1–2: 67–74. 10.1016/j.mrfmmm.2008.12.010 19162045

[77] Byrns MC, Duan L, Lee SH, Blair IA, Penning TM (2010) Aldo-keto reductase 1C3 expression in MCF-7 cells reveals roles in steroid hormone and prostaglandin metabolism that may explain its over-expression in breast cancer. J Steroid Biochem Mol Biol 118;3: 177–187. 10.1016/j.jsbmb.2009.12.009 20036328 PMC2819162

[78] Khanim F, Davies N, Velica P, Hayden R, Ride J, et al (2014) Selective AKR1C3 inhibitors do not recapitulate the anti-leukaemic activities of the pan-AKR1C inhibitor medroxyprogesterone acetate. Br J Cancer 110: 1506–1516. 10.1038/bjc.2014.83 24569460 PMC3960632

[79] Gorman A, McGowan A, Cotter TG (1997) Role of peroxide and superoxide anion during tumour cell apoptosis. FEBS Lett 404;1: 27–33. 9074631 10.1016/s0014-5793(97)00069-0

[80] Baird TD, Wek RC (2012) Eukaryotic initiation factor 2 phosphorylation and translational control in metabolism. Adv Nutr 3;3: 307–321. 10.3945/an.112.002113 22585904 PMC3649462

